# Radiological characteristics and injury mechanism of Logsplitter injury: a descriptive and retrospective study

**DOI:** 10.1186/s12891-024-07688-4

**Published:** 2024-07-26

**Authors:** Jing-Qi Liang, Yan Zhang, Yang Yue, Hui Feng, Pei-Long Liu, Xiao-Jun Liang, Hong-Mou Zhao

**Affiliations:** Foot and Ankle Surgery Department, Honghui Hospital of Xi’an Jiaotong University, No. 76 Nanguo Road, Xi’an 710054, China

**Keywords:** Ankle injuries, Ankle fracture dislocation, Logsplitter injury, Mechanism, Radiology

## Abstract

**Background:**

Logsplitter Injury is a type of high-energy ankle fracture dislocation. The mechanism of injury has not been described in detail. A detailed understanding of the radiological features and pathological changes can further guide treatment.

**Methods:**

Between April 2009 and December 2018, a retrospective analysis was conducted on 62 patients with Logsplitter injury. The study analysed the characteristics of fibular injury, tibial injury, syndesmosis injury, medial injury and lateral ligament injury on preoperative X-ray and CT scans. The incidence of the different injury types was summarised. The correlation between Logsplitter injuries and the mechanisms causing them were analysed using the Lauge-Hansen classification of ankle fractures.

**Results:**

The study provides data on the types of fractures observed. Of the total fractures, 98.4% were open fractures. The fibula injuries were classified as no fracture (1.6%), transverse or short oblique fractures (61.3%), butterfly fragments (25.8%), and comminuted fractures (11.3%). The tibial injuries included compression of lateral articular surfaces (38.7%) and posterior compressions (6.5%). Medial injuries, including medial malleolar fractures, accounted for 87.1%, and deltoid ligament rupture accounted for 12.9%. The study found that injuries to the syndesmosis consisted of simple ligament ruptures (11.3%), Tillaux fractures (8.1%), Volkmann fractures (43.5%), and Tillaux and Volkmann fractures (37.1%). In 12.9% of cases, there was a complete rupture of the lateral collateral ligament. Based on the Lauge-Hansen classification, 87.1% of injuries were pronation-abduction injuries, while 8.1% were pronation and external rotation injuries, and 1.6% were supination external rotation injuries. Furthermore, 3.2% of cases could not be classified.

**Conclusion:**

The pathoanatomic characteristics of Logsplitter injury are diverse, with some cases accompanied by collateral ligament injury. It is important to note that these evaluations are objective and based on current results. The most common injury mechanism is vertical violence combined with abduction, although in some cases, it may be a vertical combined external-rotation injury.

**Level of evidence:**

(4) case series.

**Trial registration:**

This study has been approved by the ethical research committee of the Honghui Hospital of Xi’an Jiaotong University, under the code: 202,003,002.

## Background

Ankle fracture is the most common type of intra-articular fracture involving the lower limbs, accounting for approximately 9% of systemic fractures [1–3]. It is caused primarily by torsional violence, some of which can occur after vertical violence. The vast majority of ankle fractures are low-energy injuries, which can involve the bony structure around the ankle and ligament structures and are rarely accompanied by ankle dislocation. In this type of fracture, some patients may have separation of the distal tibiofibular syndesmosis. This is usually due to low-energy rotational forces. Meanwhile, it may also be attributed to high-energy axial vertical forces or concurrent rotational forces, accompanied by lateral dislocation of the talus and proximal displacement into the distal tibiofibular space [4].

This particular type of ankle fracture with dislocation has been categorized as Logsplitter injury, as defined by Bibel et al. in 2014 [4]. However, there are few reports on Logsplitter injury and controversy still exists regarding the exact injury mechanism, when observing the radiologic manifestations of this type of injury, orthopedic surgeons often mistake it for a pilon fracture caused by vertical violence. However, it is actually quite different from the traditional pilon fracture [5]. The radiological characteristics of the injuries at different sites were not described in detail. Consequently, there are different opinions about the understanding and treatment of such injuries. Critically, for bone joint trauma, a full understanding of its injury mechanism and the detailed injury characteristics is of great significance for comprehending the disorder and formulating reasonable therapeutic plans. Accordingly, in our study, a retrospective retrieval was carried out for cases of ankle fracture in the last 10 years, with corresponding preoperative radiological data of Logsplitter injury collected for statistical analysis. This review is intended to summarize the radiological findings of Logsplitter injury during a high-energy ankle fracture and the pathological injury characteristics of the anatomical structures around the ankle. Meanwhile, the mechanism of Logsplitter injury was also discussed according to its radiological findings.

## Materials and methods

### Study design

In this study, we reviewed 3,875 cases of ankle fractures with dislocations previously treated in our foot and ankle surgery department and screened for cases consistent with Logsplitter injury, analysis includes including fibular injury, tibial injury, distal tibiofibular syndesmosis injury, medial ankle injury and lateral ligament injury, and the assessment of lateral ankle ligament injury was based on the compatibility of the talofibular joints. The proportions of different ankle fracture types in Logsplitter injury were analyzed by integrating them with the Lauge-Hansen classification of ankle fracture, and analysed the injury characteristics of different types in patients with Logsplitter injury.

### Inclusion and exclusion criteria

**Fig. 1:**
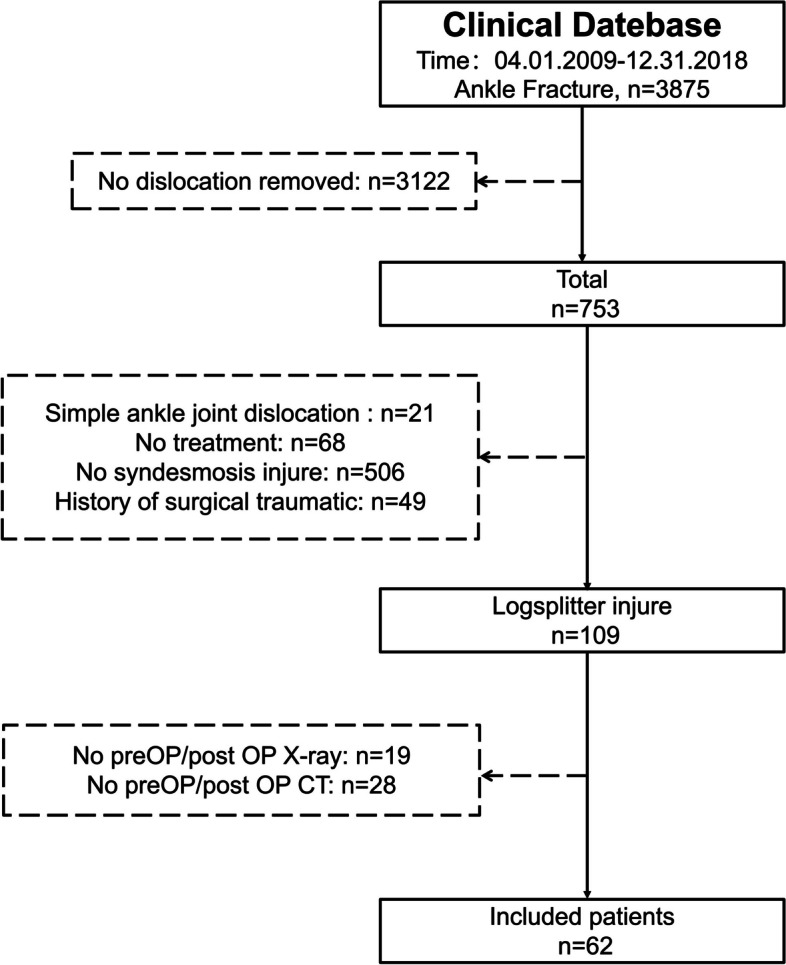
Patient selection

Inclusion criteria: (1) patients aged ≥ 18 years old at the time of injury; (2) patients with complete preoperative X-ray and CT data; and (3) The patient's injury meets radiologic criteria for a Logsplitter injury, which involves a fracture-dislocation of the ankle with proximal displacement of the talus beyond the level of the distal tibial articulation to the lower syndesmosis [4]. The exclusion criteria were as follows: (1) patients with pathological fracture; (2) patients with a congenital deformity of the ankle; and (3) patients who had a definite surgical trauma history of the ankle before this injury (Fig. 1). This study was approved by the ethical research committee of the Honghui Hospital of Xi’an Jiaotong University. (Approval No. 201806008).

### Extraction of clinical and radiological data

The radiological data for all patients was collected from the Picture Archiving and Communication System (PACS). Two senior radiologists jointly reviewed the data and extracted it after reaching a consistent diagnosis. Data were retrieved for extracting basic information, injury causes, medical history, physical examination, and other patient information.

### Radiological characteristics acquisition and analysis

Based on the anteroposterior and lateral X-ray films and preoperative CT data of the patients' injured ankles before reduction, two residents in our department evaluated the relevant radiological data according to the research design. In cases of inconsistent evaluation results, the senior associate chief physicians of our department were consulted for re-evaluation to reach an agreement after discussion. The X-ray films of all included patients were classified using the Lauge-Hansen classification by two residents. Cases with inconsistent classifications were re-evaluated by the associate chief physicians, and their opinions on consistency were included after discussion.

### Statistical analysis

The incidence of each injury characteristic was calculated with Excel 2017 software (Microsoft Office, Microsoft Corporation, USA), and the results are presented as percentages (100%).

## Results

A total of 62 patients with Logsplitter injury were enrolled in this study, accounting for 1.6% (62/3,875) of all cases with ankle fractures. The cases included 44 men and 18 women aged between 21 and 69 years (mean age 42.3 ± 11.7 years). Of these cases, 27 affected the left side and 35 affected the right side. In terms of the causes of injury, there were 37 cases of falling injuries, 16 cases of traffic accident injuries, 6 cases of injuries caused by stepping down steps or from a bus, and 3 cases of sports-related injuries. Additionally, there were 61 cases of open fractures and 1 case of a closed fracture.

**Fig. 2:**
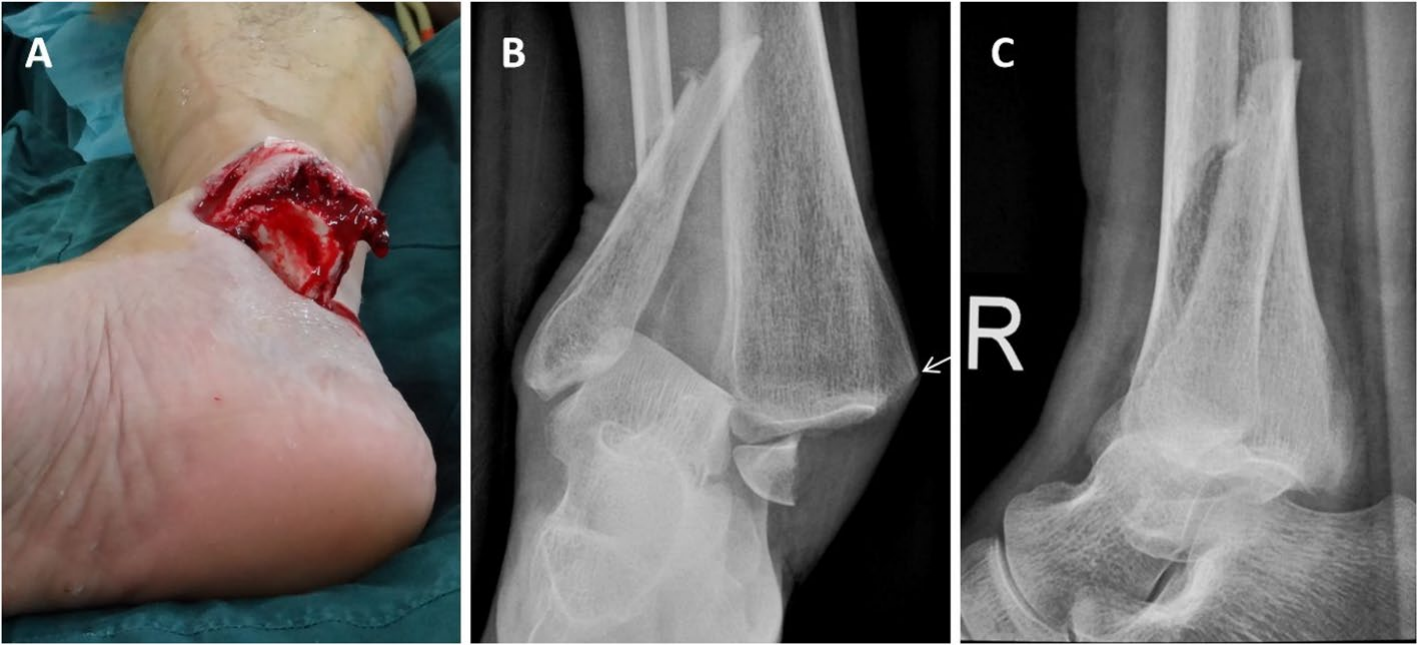
**A** Medial open wound of the ankle joint and bone exposure of the distal tibia. **B**,**C** Anteroposterior and lateral X-ray films of the ankle with pronation-external rotation closed Logsplitter injury, with the visible continuous and complete medial soft tissue of the ankle joint displayed on the anteroposterior X-ray film (arrow)

**Fig. 3:**
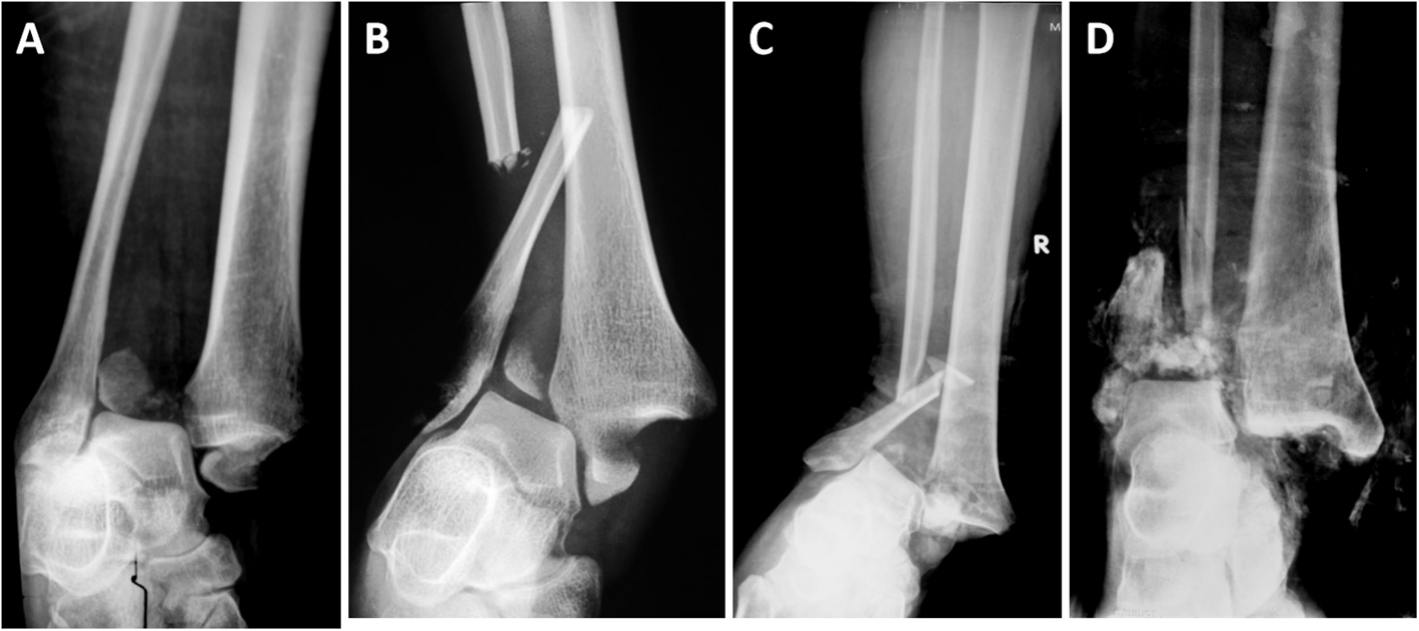
Characteristics of the Fibula injury. **A** No fracture of the fibula. **B** High transverse fracture of fibula. **C** Fibular fracture accompanied by a butterfly fragmen. **D** Comminuted fracture of the fibula, with the fracture located at or below the level of the distal tibiofibular syndesmosis

**Fig. 4:**
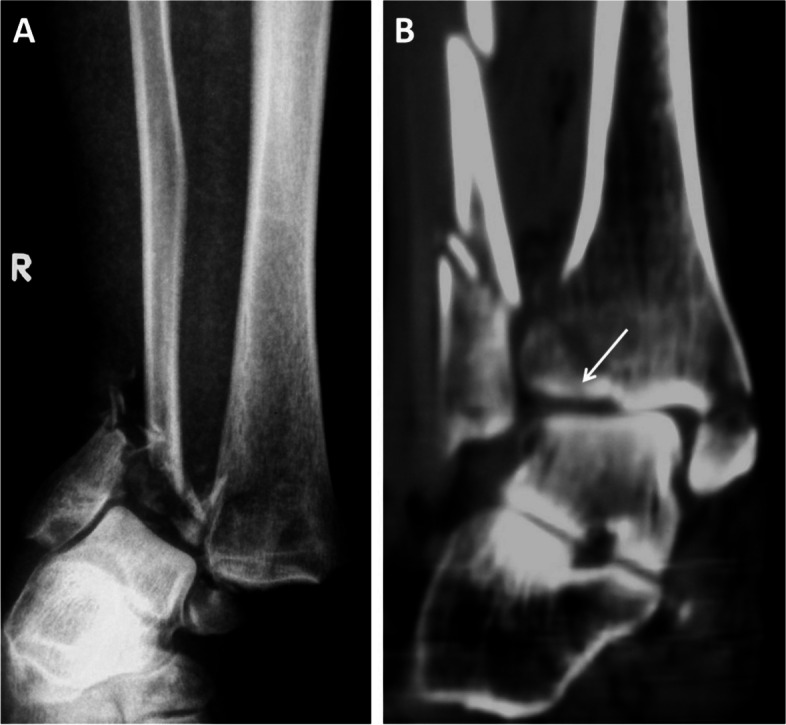
Characteristics of the tibial injury. **A** The talus was displaced to the syndesmosis, talofibular joint congruence. **B** Compression fracture of the lateral articular surface of the distal tibia displayed by CT of the right ankle joint (arrow)

**Fig. 5:**
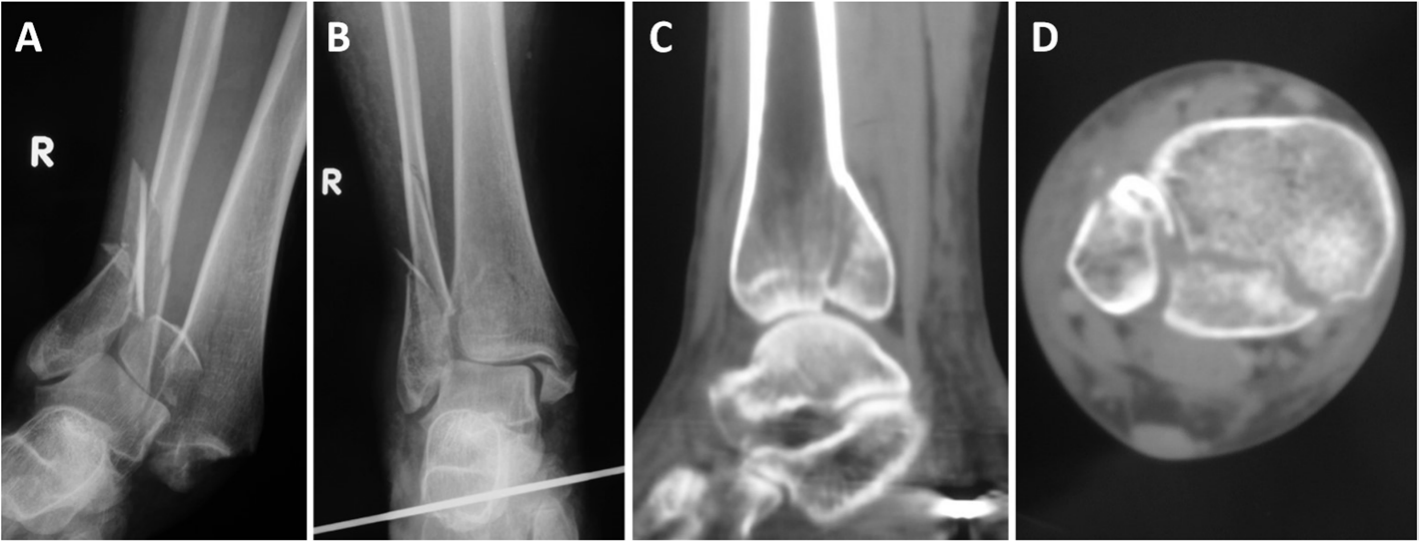
Open Logsplitter injury of the right ankle joint. **A**,**B** Anteroposterior X-ray film of the right ankle before and after reduction. **C**,**D** Logsplitter injury combined with posterior compression fracture of the distal tibia as displayed by CT of the right ankle joint (posterior Pilon fracture)

**Fig. 6:**
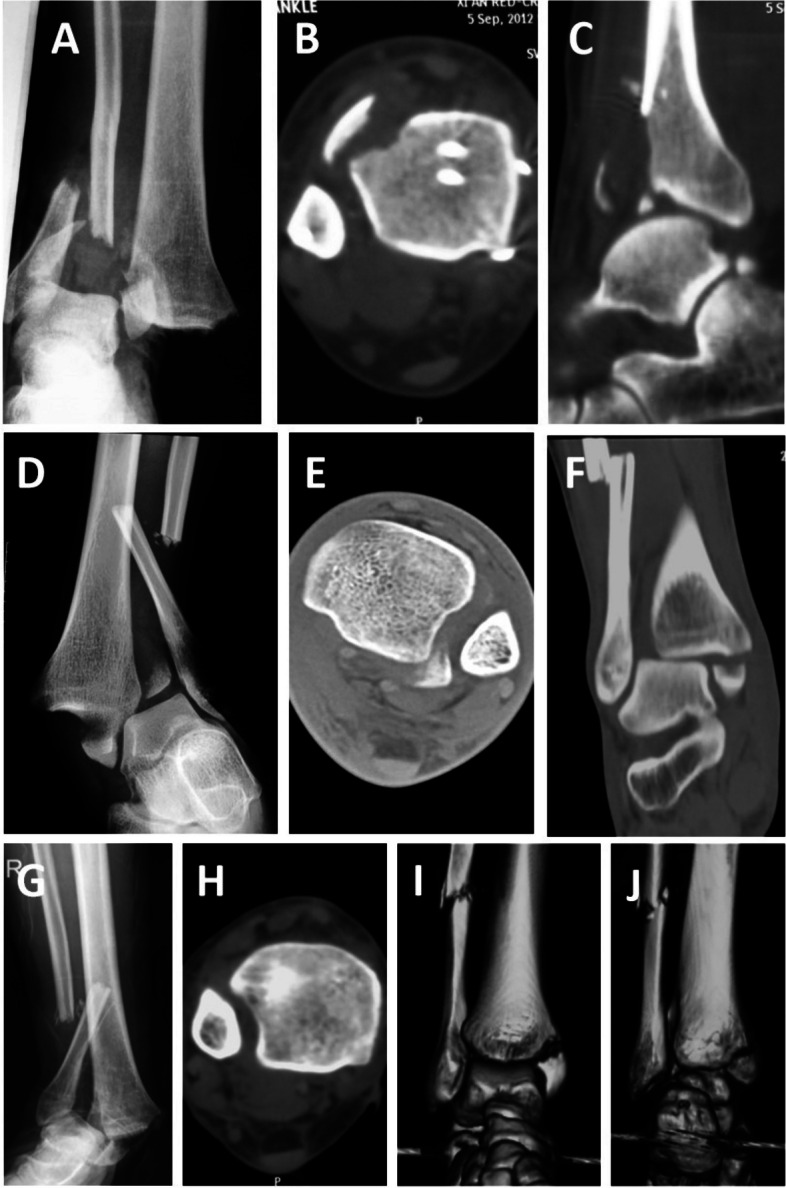
Characteristics of distal tibiofibular syndesmosis injuries. **A**,**B**,**C** Separation of the distal tibiofibular syndesmosis combined with a simple Tillaux avulsion fracture as displayed by the anteroposterior X-ray and CT of the ankle joint. **D**,**E**,**F** Separation of the distal tibiofibular syndesmosis combined with the simple Volkmann avulsion fracture as displayed by the anteroposterior X-ray and CT of the ankle joint. **G**,**H**,**I**,**J** Simple separation of the distal tibiofibular syndesmosis as displayed by the anteroposterior X-ray and CT of the right ankle joint

**Fig. 7:**
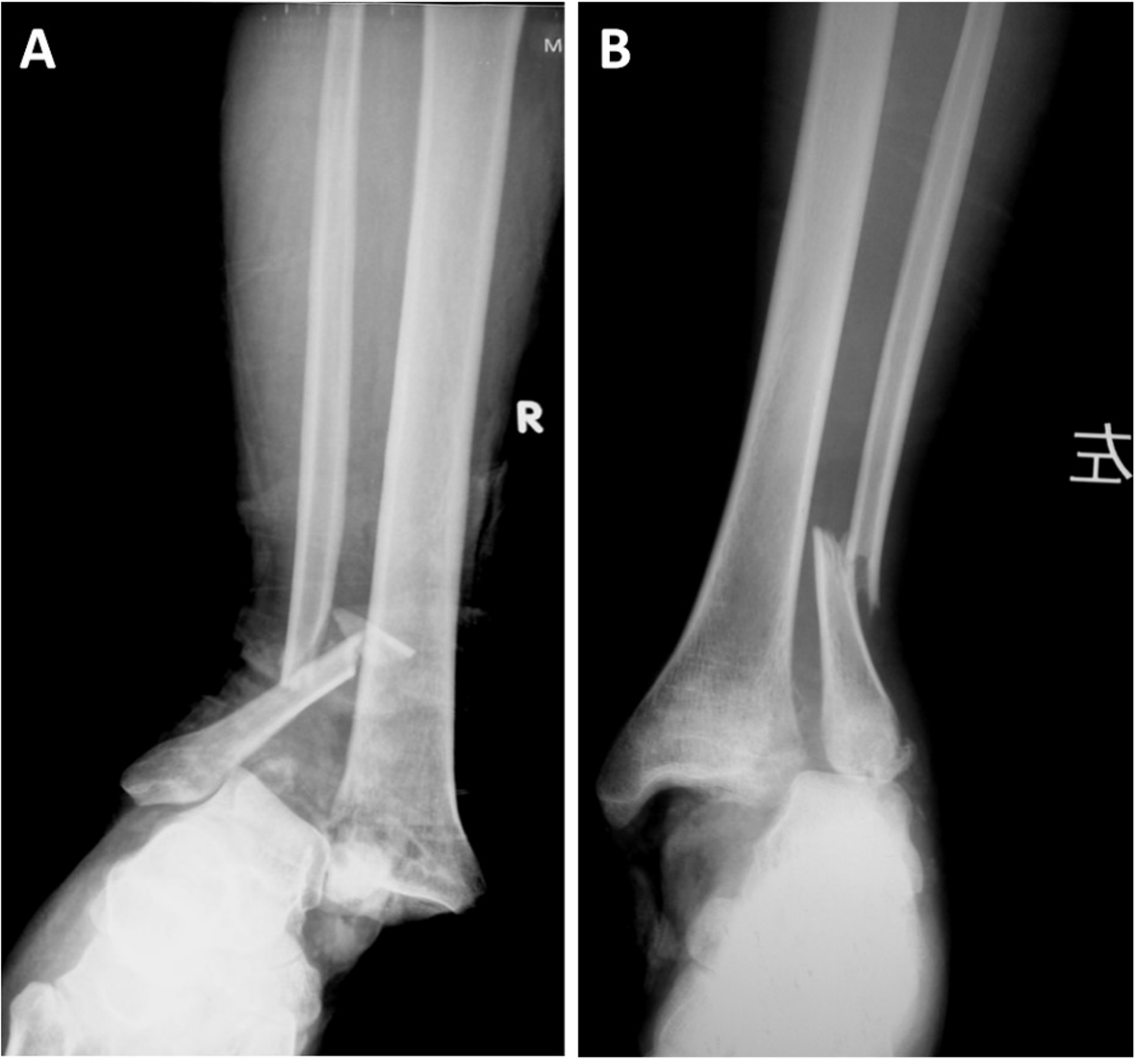
Characteristics of medial injury in patients with open Logsplitter injury of the ankle joint. **A** An avulsion fracture of the medial malleolus and talofibular joint congruence by anteroposterior X-ray film of the right ankle. **B** Deltoid ligament rupture and dislocation of the talofibular joint by anteroposterior X-ray film of the left ankle joint

Among all cases with Logsplitter injuries, open fractures accounted for the largest proportion of 98.4% (61/62), all of which were open wounds on the medial ankle, as shown in Fig. 2. The injury characteristics and the incidence of the different injury types in Logsplitter injuries were analyzed based on the radiological data. Regarding the characteristics of fibular injury, there were no fractures in 1.6% (1/62), transverse or short oblique fractures in 61.3% (38/62), butterfly bone fragments in 25.8% (16/62) and comminuted fractures in 11.3% (7/62); 14.5% (9/62) of the cases had fracture lines below the level of the distal tibiofibular syndesmosis, and 83.9% (52/62) had fractures above the level of the distal tibiofibular syndesmosis (Fig. 3). Meanwhile, in terms of the characteristics of the tibial injury, 38.7% (24/62) were compression fractures of the lateral articular surface of the distal tibia (Fig. 4), 6.5% (4/62) were combined with posterior compression fractures (posterior Pilon fractures) (Fig. 5), and no compression fractures of the anterior articular surface of the tibia (0%) were observed. The characteristics of the distal tibiofibular syndesmosis injury included simple distal tibiofibular ligament fracture 11.3% (7/62), concurrent Tillaux avulsion fracture 8.1% (5/62), concurrent Volkmann avulsion fracture 43.5% (27/62), and concurrent Tillaux and Volkmann avulsion fracture 37.1% (23/62) (Fig. 6). In addition, the characteristics of the medial injury included avulsion fracture of the medial malleolus (87.1%, 54/62) and rupture of the deltoid ligament (12.9%, 8/62). Among the enrolled cases, 12.9% (8/62) had a lateral injury combined with dislocation of the talofibular joints, all of which were complicated by rupture of the deltoid ligament (Fig. 7).

According to the Lauge-Hansen classification, 87.1% (54/62) of cases were confirmed to have pronation-abduction ankle injury, 8.1% (5/62) showed ankle pronation-external rotation, 1.6% (1/62) had supination-external rotation (Fig. 5), and the classification failed in 3.2% (2/62) of the enrolled cases.

## Discussion

### Characteristics of Logsplitter injury

Logsplitter injury is a special type of ankle fracture and dislocation due to strong forces and it has various characteristics. At present, there are few reports on the radiological characteristics of Logsplitter injury less reported internationally, and there is a lack of unified consensus on its mechanism of injury. As described previously, Bibel [6] proposed and defined Logsplitter injury for the first time. It manifests as an ankle fracture and dislocation with the talus displaced into the distal tibiofibular joint space on radiology, similar to the "wedge splitter"-like injury. According to their report concerning the injury characteristics and incidence, our study has provided a more comprehensive description of the radiological characteristics of Logsplitter injury as well as the corresponding incidence of different injury characteristics in the enrolled cases. Specifically, there was a relatively higher incidence of transverse or short oblique fibular fractures in our reported data, with a similar incidence of avulsion fracture of the medial malleolus and compression fracture of the distal tibia when compared with the data provided by Bibel. In this study, there is an interesting finding: when the medial injury is an avulsion fracture, neither the talofibular joint nor the lateral collateral ligament is injured; when the medial injury is a deltoid ligament rupture, the talofibular joint is also dislocated, this may be related to the damage mechanism (Fig. 7). In the past, surgeons have generally given little thought to re-treating lateral ankle ligament injuries after fixation of the fracture in the surgical treatment of ankle fracture-dislocations, however, for cases such as the one presented in Fig. 7B, repair of the lateral collateral ligament should actually be considered when the fracture has been managed. Depending on the different radiologic presentations, better decisions can be made preoperatively, rather than following the past by simply treating the fracture. This is the main significance of this study.

### Mechanism of injury

According to the report by Wang [7], injured patients could be classified into typical and atypical groups according to the injury mechanism and the degree of talus displacement to the distal tibiofibula. Specifically, in their study, there were 19 cases of typical injuries, which were mainly caused by vertical axial trauma, including pronation-external rotation in 10 cases, pronation-abduction in 7 cases, supination-external rotation in 1 case and supination-adduction in 1 case. Meanwhile, there were 22 cases of atypical injury primarily induced by rotational violence and then talar displacement due to a vertical force, resulting in incomplete separation of the distal tibiofibular, without injury or with only mild injury to the ligament. Among these 22 cases, pronation-external rotation was observed in 6 cases, pronation-abduction in 14 cases and supination-external rotation in 2 cases. In our study, it was believed that Logsplitter injury was the result of lateral displacement of the talus, which was mostly explained by pronation external rotation/abduction, opposite to supination adduction.

Prior research has compared the clinical curative effect and prognosis of open and closed Logsplitter injury [8]. Our corresponding data revealed that among the 15 cases of open injury, 12 cases were complicated with deltoid ligament injury, 9 cases with fibular fracture, 10 cases with medial malleolar fracture and 9 cases with tibial Pilon fracture. In the other 21 cases of closed injury, 12 cases were complicated with deltoid ligament injury, 12 cases with fibular fracture, 9 cases with medial malleolar fracture and 1 case with tibial Pilon fracture. The incidence of fibular fracture was approximately 60% in both injuries. Moreover, in the 21 patients with closed Logsplitter injury in that study, the ankle joint width doubled after displacement of the talus to the distal tibiofibular joint under a high-energy violence. In addition, there is thin coverage of the soft tissues around the joint, and the incidence of closed injury is extremely low. Only 1 case of closed injury was reported in our study, accompanied by medial skin necrosis. In our opinion, under a high-energy abduction-external rotation force, the talus enters the distal tibiofibular space, and the specific incidence of injury with fibular integrity is quite low due to the high-energy violence.

In cadaveric studies, the researchers established the method of Lauge-Hansen classification [9–12]. In their studies, reconstruction of ankle fractures was simulated based on the foot position (supination/pronation) and the direction of the force (adduction/abduction/external rotation). In 1990, Marco [13] reported a case of Logsplitter injury and considered pronation-external rotation as the injury mechanism of this type of fracture. In our study, according to the radiological data of fracture types in all patients, 87.1% were pronation-abduction and 8.1% were pronation-external rotation. In our opinion, a simple vertical violence results in a Pilon fracture. The results of this study revealed that Logsplitter injury was primarily a result of a rotational violence, which occurred with/without vertical forces. When the foot was in the pronation position, Logsplitter injury might have developed from strong abduction or external rotation forces.

### Logsplitter injury treatment and prognosis

The radiological manifestations of Logsplitter injury with different characteristics can provide a comprehensive understanding of its injury characteristics, exhibiting guiding significance in surgical design and the choice of internal fixation. In the case of simultaneous lateral articular surface compression, intraoperative reduction and bone grafting are required to avoid secondary valgus deformity postoperatively. Meanwhile, when there is a concurrent posterior Pilon fracture, fixation with screws alone may have difficulty effectively resisting axial sliding [14–17]. In this case, prolonged postoperative braking may be needed to avoid displacement, and a firmer effect can be realized by using buttress plate fixation via a posterior approach, which can facilitate an earlier recovery of functional exercise after operation [5, 18]. The reduction of the fibula is of great significance for an injury to the distal tibiofibular syndesmosis [19]. In Logsplitter injury, the emphasis should be placed on the fibula length and rotation reduction, which is critical for the recovery of ankle function in the later stages [20]. Otherwise, poor reduction of the fibula may induce widening of the tibiotalar joint space and lateral dislocation of the talus, which can eventually lead to traumatic ankle arthritis [21]. It is currently considered that timely surgery should be adopted when there is a separation of the distal tibiofibular syndesmosis, and the reliability of the reduction may determine the postoperative effect [22, 23].Unnecessary surgery can lead to catastrophic outcomes, such as ankle fusions [24].

In this study, all patients with Logsplitter injury showed complete separation of the distal tibiofibular syndesmosis, and injury of the distal tibiofibular syndesmosis accounted for 1% ~ 11% of all cases with ankle injuries [25]. In their research, it was believed that there were differences in injury energy, soft tissue injury and traumatic arthritis between Logsplitter injury and Pilon fracture or traditional ankle fracture [7]. The postoperative AOFAS score of Logsplitter injury was merely 67.0 ± 26.8 points, as reported by Beble [4]. Despite the controversy regarding the mechanism and prognosis of this injury, the complexity of the injury and the severity of joint damage may predict a worse clinical outcome than that of a simple ankle fracture. Therefore, there may be a greater challenge in the reasonable treatment of this injury by surgeons in clinical practice.

## Conclusions

In summary, Logsplitter injury is an ankle fracture with dislocation caused by high-energy forces. Its mechanism of injury may be predominated by an abduction-vertical violence, which can be partially explained by external rotation-vertical forces as well. It is characterized by diverse radiological characteristics, with serious soft tissue injury and frequent compression of the distal tibial articular surface. Its radiological manifestations can be summarized. The main limitation of this study is the relatively small sample size, which may be attributed to the relatively low incidence of Logsplitter injury. Our future research will improve the radiological characteristics database of this injury on the basis of an expanded sample size.

Analysis of its radiological characteristics in this study revealed that the major fracture type of Logsplitter injury is dominated by pronation-abduction based on the Lauge-Hansen classification, a few of which are in accordance with the characteristics of pronation-external rotation and supination-external rotation injury. However, the most important difference from the Lauge-Hansen classification is that when the deltoid ligament is ruptured, a fracture of the fibula with rupture of the lateral collateral ligament often occurs, and open reduction and internal fixation may be required along with concomitant repair of the lateral collateral ligament, preoperative evaluation of the radiological manifestations of the injury is of guiding significance for surgical design in the clinical setting.

## Data Availability

The datasets used and/or analyzed during the current study are available from the corresponding author on reasonable request.

## References

[1] Leininger RE, Knox CL, Comstock RD. Epidemiology of 1.6 million pediatric soccer-related injuries presenting to US emergency departments from 1990 to 2003. Am J Sports Med. 2007;35(2):288–93. 17092927 10.1177/0363546506294060

[2] Mandi DM, Nickles WA, Mandracchia VJ, Halligan JB, Toney PA. Ankle fractures. Clin Podiatr Med Surg. 2006;23(2):375–422. 16903159 10.1016/j.cpm.2006.02.001

[3] Sporer SM, Weinstein JN, Koval KJ. The geographic incidence and treatment variation of common fractures of elderly patients. J Am Acad Orthop Surg. 2006;14(4):246–55. 16585366 10.5435/00124635-200604000-00006

[4] Bible JE, Sivasubramaniam PG, Jahangir AA, Evens JM, Mir HR. High-energy trans-syndesmotic ankle fracture dislocation-the “Logsplitter” injury. J Orthop Trauma. 2014;28(4):200–4. 24177591 10.1097/01.bot.0000435605.83497.53

[5] Biz C, Angelini A, Zamperetti M, Marzotto F, Sperotto SP, Carniel D, Lacobellis C, Ruggieri P. Medium-long-term radiographic and clinical outcomes after surgical treatment of intra-articular tibial pilon fractures by three different techniques. Biomed Res Int. 2018;1(2018):6054021. 10.1155/2018/6054021 PMC5852840 29687005

[6] Day GA, Swanson C, Hulcombe BG. Operative treatment of ankle fractures: a minimum ten-year follow-up. Foot Ankle Int. 2001;22(2):102–6. 11249218 10.1177/107110070102200204

[7] Wang Z, Tang X, Li S, Wang XH, Gong LF, Zhong T, Wang KZ. Treatment and outcome prognosis of patients with high-energy transsyndesmotic ankle fracture dislocation-the “Logsplitter” injury. J Orthop Surg Res. 2017;12(1):3. 28073376 10.1186/s13018-016-0502-y PMC5223364

[8] Ren Y, Wu SZ, Deng W, Song R, Dong HX, Li YX, Chen Y, Liu Y, Huang FG, Zhang H. Effectiveness comparison of open reduction and internal fixation for open and closed ankle logsplitter fractures. Zhongguo Xiu Fu Chong Jian Wai Ke Za Zhi. 2018;32(10):1302–7. 30215494 10.7507/1002-1892.201712073 PMC8414161

[9] Boszczyk A, Fudalej M, Kwapisz S, Klimek U, Maksymowicz M, Kordasiewicz B, Rammelt S. Ankle fracture - Correlation of Lauge-Hansen classification and patient reported fracture mechanism. Forensic Sci Int. 2018;282:94–100. 29182957 10.1016/j.forsciint.2017.11.023

[10] Lauge-Hansen N. Fractures of the ankle. II. Combined experimental-surgical and experimental-roentgenologic investigations. Arch Surg. 1950;60:957–85. 10.1001/archsurg.1950.01250010980011 15411319

[11] Lauge-Hansen N. Fractures of the ankle. III. Genetic roentgenologic diagnosis of fractures of the ankle. Am J Roentgenol Radium Ther Nucl Med. 1954;71:456–71. 13124631

[12] Okanobo H, Khurana B, Sheehan S, Duran-Mendicuti A, Arianjam A, Ledbetter S. Simplified diagnostic algorithm for lauge-hansen classification of ankle injuries. Radiographics. 2012;32(2):E71-84. 22411951 10.1148/rg.322115017

[13] Molinari M, Bertoldi L, De March L. Fracture dislocation of the ankle with the fibula trapped behind the tibia. Acta Orthop. 1990;61(5):471–2. 10.3109/17453679008993567 2239178

[14] Davidovitch RI, Elkataran R, Romo S, Wslsh M, Egol KA. Open reduction with internal fixation versus limited internal fixation and external fixation for high grade pilon fractures (OTA Type 43C). Foot Ankle Int. 2011;32(10):955–61. 22224324 10.3113/FAI.2011.0955

[15] Zhao HM, Li Y, Liang XJ. Different internal fixation methods in treatment of posterior ankle joint injuries: a biomechanic study. Chin J Bone Joint Injury. 2016;31(6):613–6.

[16] Jansen H, Fenwick A, Doht S, Frey S, Meffert R. Clinical outcome and changes in gait pattern after pilon fractures. Int Orthop. 2013;37(1):51–8. 23229797 10.1007/s00264-012-1716-1 PMC3532654

[17] Ketz J, Sanders R. Staged posterior tibial plating for the treatment of orthopaedic trauma association 43C2 and 43C3 tibial pilon fractures. J Orthop Trauma. 2012;26(6):341–7. 22207206 10.1097/BOT.0b013e318225881a

[18] Naqvi GA, Cunningham P, Lynch B, Galvin R, Awan N. Fixation of ankle syndesmotic injuries: comparison of TightRope fixation and syndesmotic screw fixation for accuracy of syndesmotic reduction. Am J Sports Med. 2012;40(12):2828–35. 23051785 10.1177/0363546512461480

[19] Teramoto A, Kura H, Uchiyama E, Suzuki D, Yamashita T. Three-dimensional analysis of ankle instability after tibiofibular syndesmosis injuries: a biomechanical experimental study. Am J Sports Med. 2008;36(2):348–52. 17940143 10.1177/0363546507308235

[20] Lamothe J, Baxter JR, Gilbert S, Murphy CI, Karnovsky SC, Drakos MC. Effect of complete syndesmotic disruption and deltoid injuries and different reduction methods on ankle joint contact mechanics. Foot Ankle Int. 2017;38(6):694–700. 28298142 10.1177/1071100717696360

[21] Noh JH, Roh YH, Yang BG, Kim SW, Lee JS, Oh MK. Outcomes of operative treatment of unstable ankle fractures: a comparison of metallic and biodegradable implants. J Bone Joint Surg Am. 2012;94(22): e166. 23172333 10.2106/JBJS.K.01221

[22] Chissell HR, Jones J. The influence of a diastasis screw on the outcome of Weber type-C ankle fractures. J Bone Joint Surg B. 1995;77(3):435–8. 10.1302/0301-620X.77B3.7744931 7744931

[23] Xu HL, Li X, Zhang DY, Fu ZG, Wang TB, Zhang PX, Jiang BG, Shen HL, Wang G, Wang GL, Wu XB. A retrospective study of posterior malleolus fractures. Int Orthop. 2012;36(9):1929–36. 22777382 10.1007/s00264-012-1591-9 PMC3427438

[24] Biz C, Hoxhaj B, Aldegheri R, Lacabellis C. Minimally invasive surgery for tibiotalocalcaneal arthrodesis using a retrograde intramedullary nail: preliminary results of an innovative modified technique. J Foot Ankle Surg. 2016;55(6):1130–8. 27524730 10.1053/j.jfas.2016.06.002

[25] Yu GR, Chen DW, Zhao HM. Treatment outcomes of buttress plating in treatment of posterior pilon fractures. Chin J Traum. 2013;29(3):243–8.

